# Caregiving in severe mental illness: the psychometric properties of the Involvement Evaluation Questionnaire in Portugal

**DOI:** 10.1186/1744-859X-11-8

**Published:** 2012-03-28

**Authors:** Manuel Gonçalves-Pereira, Bob van Wijngaarden, Miguel Xavier, Ana L Papoila, José M Caldas-de-Almeida, Aart H Schene

**Affiliations:** 1 Department of Mental Health, CEDOC, Faculdade de Ciências Médicas, Universidade Nova de Lisboa, Lisbon, Portugal; 2 The Netherlands Institute of Mental Health and Addiction, Utrecht, The Netherlands; 3 Department of Biostatistics and Informatics, Faculdade de Ciências Médicas, Universidade Nova de Lisboa, and CEAUL, Lisbon, Portugal; 4 Department of Psychiatry, Academic Medical Center, University of Amsterdam, Amsterdam, The Netherlands

**Keywords:** Caregiver, crosscultural psychiatry, family member, measurement validation, questionnaires, schizophrenia

## Abstract

**Background:**

Despite the achievements of previous research, caregiving assessments in severe mental illness should be crossculturally validated in order to define risk groups or to evaluate family work. This study reports on the psychometric properties of the European version of the Involvement Evaluation Questionnaire (IEQ-EU) in Portugal.

**Methods:**

A Portuguese translation of the IEQ-EU was developed according to the 'European Psychiatric Services: Inputs Linked to Outcome Domains and Needs' (EPSILON) group guidelines. We then studied 194 caregivers who were related to patients with schizophrenia spectrum disorders in psychiatric outpatient services. All relatives were assessed using the IEQ-EU. In order to describe the corresponding patients' sample, the majority (n = 162) was evaluated with the World Health Organization Disability Assessment Schedule (WHO-DAS II); 108 patients were also assessed with the Brief Psychiatric Rating Scale (BPRS) and the Global Assessment of Functioning (GAF).

**Results:**

The factor structure of the Portuguese version of the questionnaire was similar to the original; internal consistency was good, with Cronbach's α ranging from 0.71 to 0.87 in the IEQ-EU scales (total score and domains: tension, supervision, worrying, urging); test-retest reliability yielded intraclass correlation coefficients (ICCs) from 0.80 to 0.94, concerning the same scores. Ecological validity was confirmed. Most caregiving consequences were reported on the worrying domain of the IEQ-EU.

**Conclusions:**

Validity and reliability of the Portuguese IEQ-EU translation were established. Specifically the four IEQ-EU subscale domains seem to be valid in Portugal.

## Introduction

Despite huge amounts of high-quality research related to caregiving in severe mental illness (see for example [1-4]), a need exists for the crosscultural validation of caregiver instruments, and for the development of national norms regarding assessments. Without better knowledge of the intercultural validity of measures and local standards, international comparisons will be undermined by doubts regarding the origin of differences between scorings (for example, sampling vs real cultural differences) [5].

The European Psychiatric Services: Inputs Linked to Outcome Domains and Needs (EPSILON) study was a crossnational, cross-sectional survey [6,7], which compared characteristics, needs and quality of life of people with schizophrenia and their caregivers in five European countries (Denmark, England, Italy, The Netherlands and Spain). Standardized versions of related key research instruments were produced, including the Involvement Evaluation Questionnaire (IEQ) for the assessment of caregiving consequences [8].

The IEQ measures the consequences of psychiatric disorders for relatives, friends or other significant persons involved, being a well established tool for assessments of caregiving impact in psychotic, mood or mixed disorders [5,9]. In the EPSILON study the English, Danish, Italian and Spanish translations were validated [8]. Over the last decade ten more translations were made. Validation data on the German, Swedish, Malawi, Chinese, Polish and Arabic versions are available [10-15]. The Finnish, French and Greek versions have not been validated yet [16]. A previous version of the IEQ was used in Portugal [17], but to date the IEQ-EU has not been adequately validated in Portuguese populations.

Therefore, our aim was to validate and test the reliability of a Portuguese translation of the IEQ-EU, according to the EPSILON study methodology, in Portuguese caregivers of psychotic outpatients. In this paper, we document the validity of the IEQ-EU scales in Portugal (focusing on ecological, face value and content aspects), along with their test-retest reliability and internal consistency.

## Methods

### Study design and participants

After the development of the IEQ-EU Portuguese translation and pretests, factor structure and internal consistency were determined.

We studied a non-randomized sample of primary caregivers of outpatients with chronic psychosis, who were in contact with mental health services in Lisbon (n = 194). These services were Hospital S. Francisco Xavier, a public hospital, and Clínica Psiquiátrica de S José, a non-profit facility. The majority of participants (n = 108) formed the baseline sample of the 'FAmilies of people with PSychotic disorders' (FAPS) survey, for which preliminary results have been presented [18]. In this paper, only the IEQ-EU data will be used. Results for the FAPS baseline study are reported in detail elsewhere [19] (Gonçalves-Pereira M, Xavier M, van Wijngaarden B, Papoila AL, Schene AH, Caldas de Almeida JM: Impact of psychosis in a Portuguese population: a cross-cultural exploration of burden, distress, positive aspects and clinical-functional correlates, unpublished). Test-retest reliability was evaluated in those caregivers who agreed to collaborate (n = 50), by asking them to complete the IEQ-EU a second time within a 2 to 3 week period. The overall sample size was not defined *a priori*, yet it should allow for the study of the IEQ-EU psychometrics, including factorial validity. For that purpose, the FAPS sample was enlarged with additional participants.

In the FAPS survey, 108 patients with *International Classification of Diseases, 10th edition *(ICD-10) *Diagnostic Criteria for Research *(DCR) schizophrenia (F20), schizoaffective (F25) or delusional disorder (F22), were included. Regarding all non-FAPS patients in this study (n = 86), a simple ICD-10 clinical diagnosis of one the above-mentioned disorders was the condition to be included. Cases with coexisting learning disability, organic disorders, or inpatient treatment episodes in the previous 2 months were excluded.

Primary caregivers were then approached. All of them were family to the patient, but relatives will be referred to as 'caregivers' for the remainder of this article. The protocol was approved by local ethical committees. Informed consent was obtained from all participants.

### The IEQ-EU and its Portuguese translation

The IEQ-EU is an 81-item questionnaire to be completed by any caregiver who, during the past 4 weeks, had at least 1 h a week contact with the patient. The questionnaire consists of seven modules: (1) demographics of patient and family, and general clinical data concerning the patient; (2) caregiving consequences of psychiatric disorders; (3) extra financial expenses; (4) the General Health Questionnaire, 12-item version (GHQ-12), used as a general measure of distress [20]; (5) professional help for patient's relative; (6) consequences for patient's children; (7) open question for additional remarks. Module 2 is the core module (31 items), where items refer to all kinds of encouragement and care the caregiver has to provide to the patient, to supervision of the patient's dangerous behaviors, to interpersonal problems between patient and caregiver, and to the caregiver's worrying (for example, 'how often have you worried about your relative's future?'). These items are scored on a five-point Likert scale (0 = never, 1 = sometimes, 2 = regularly, 3 = often, 4 = (almost) always), and the time frame is the prior 4 weeks. The caregiver's coping and subjective burden are also assessed in individual items. In research use, 27 of the 31 core module item scores can be summarized in a total or sum score, and 4 subscales or domains: tension (9 items), supervision (6 items), worrying (6 items), and urging (8 items). Two items (no. 29 and no. 43) load on two subscales [5,21]. 'Tension' refers to the strained interpersonal atmosphere between patient and relatives; 'supervision' to the caregiving tasks of ensuring and guarding related to, for example, the patient's intake of medicine or dangerous behaviors; 'worrying' to painful cognitions and concerns about patient's safety or future; and 'urging' to issues related to activating and motivating the patient. An overview of the core items of the IEQ-EU is given in the Results section.

The IEQ Portuguese translation was developed in accordance with international conventions and EPSILON guidelines, in order to maintain face and content validity [22]. All modules had to be translated except the GHQ-12, for which a Portuguese translation was available from the publisher [23]. A first draft concerned the translation of the English IEQ-EU by one of the authors (MGP), who also was involved in the Portuguese version of the original IEQ [17]. This translation was refined by a Dutch native Portuguese-fluent lay contributor using the Dutch IEQ-EU. A focus group involving caregivers, and a discussion group involving mental health professionals and researchers, were both conducted in order to check the quality and acceptability of the translation. Drafts were subsequently refined and a back translation was performed by a Dutch native Portuguese-fluent professional translator. When revised by one of the authors of the original tool (BvW), only minor corrections were made.

We chose not to report the GHQ-12 results here, as only FAPS participants completed this questionnaire. Moreover, these assessments are relevant to construct validation but not to define the psychometric properties related to the core IEQ-EU.

### Other measures

Patient clinical data not covered by the IEQ-EU, such as number of previous admissions, were collected in interviews.

Regarding the FAPS baseline sample, patients' symptoms were assessed with the extended (24 item) Brief Psychiatric Rating Scal*e *(BPRS) [24]. BPRS items are coded into seven categories (1 = no symptoms, to 7 = extremely severe). Patients' disability was assessed through interviewing caregivers and collecting other sources of information with the World Health Organization Disability Assessment Schedule II (DAS-II) [25], producing global evaluations from 0 ('excellent or very good adjustment') to 5 ('severe maladjustment'). Finally, the Global Assessment of Functioning (GAF) scale [26] was applied, in a continuum from 0 to 100. The DAS interview was also used for 54 patients in the non-FAPS sample, so that data on the global evaluation section were available for 162 cases. These clinical and functional assessments were made by trained research assistants, all of them mental health professionals.

### Statistical analysis

Descriptive statistics and non-parametric tests were used as required. Patient and caregiver characteristics are presented as frequencies and percentages for categorical data, and as mean or median, standard deviation (SD), range and minimum/maximum values for continuous variables. The 95% confidence intervals (CI) for the mean value were calculated whenever appropriate.

Principal component analysis was used to conduct an exploratory factor analysis on the IEQ scores. To verify the appropriateness of the factor analysis, three techniques were used for the assessment of the psychometric adequacy of the correlation matrix: (1) Bartlett's test of sphericity, evaluating the hypothesis that the correlation matrix is an identity matrix (that is, there is no correlation among the items); rejection of this hypothesis suggests that data are appropriate for factor analysis; (2) inspection of the off-diagonal elements of the anti-image correlation matrix (that contains the negatives of the partial correlation coefficients), enabling us to quantify individual measures of sampling adequacy (MSA) and to conclude that the correlation matrix is factorable whenever the absolute values of those elements are small; (3) Kaiser-Meyer-Olkin (KMO) statistic, an overall MSA that varies between 0 and 1 (high values for this statistic indicate that the data are unsuitable for factor analysis). Four factors were chosen *a priori *in order to provide comparable results with similar studies. The Varimax rotation technique with Kaiser normalization was used. It was postulated that factor loadings should be > 0.40. In case of several loadings > 0.40 in a particular item, this item would be used for more than one factor score.

For IEQ scores, test-retest reliability was assessed calculating intraclass correlation coefficients (ICCs) and 95% CI; Cronbach's α values were computed for internal consistency. Regarding correlation studies, Spearman's coefficients were used when a linear association was present between continuous variables. The significance level of α = 5% was considered. All data were entered and analyzed using SPSS for Windows V.15.0 (SPSS Inc., Chicago, IL, USA).

## Results

Demographics and clinical data regarding the patients and their primary caregivers are first described. The mean age of the patients in the total sample (n = 194) was 35.6 (SD 9.5) years. Most were male (63.4%), had a diagnosis of schizophrenia (70.1%) or other chronic psychosis (29.9%), a number of psychiatric admissions ranging from none to 16 (median 2.0), and a duration of illness ranging from 1 to 63 years (median 10.0). DAS mean (SD) scores were 2.6 (0.9) (n = 162). In the FAPS baseline sample (n = 108), BPRS mean (SD) scores were: 1.8 (0.5); and GAF mean (SD) scores were: 52.6 (13.8). These FAPS patients had ICD-10 DCR schizophrenia in the majority of cases (88.9%), mainly of the paranoid type, F20.0 (76.9%).

The corresponding caregivers' characteristics (n = 194) are shown in Table 1. Most were female (67.5%), parent of the patient (71.1%), and living with him/her (89.7%). All completed the IEQ-EU.

**Table 1:** Caregivers' demographics and caregiving arrangements

Caregiver demographic	Value
Mean (SD) age, years	57.7 (13.7); range: 18 to 86
Gender	Male: 63 (32.5%); female: 131 (67.5%)
Mean (SD) monthly net income	3.8 (1.5)
Marital status	Married: 123 (63.4%); single: 15 (7.7%); divorced: 31 (16.0%); widowed: 25 (12.9%)
Living situation	Extended family: 14 (7.2%); with spouse/children: 156 (80.4%); with parents/brothers: 14 (7.2%); with other relatives: 4 (2.1%); with others: 3 (1.5%); alone: 3 (1.5%)
Relationship to patient	Mother/father: 138 (71.1%); sibling: 24 (12.4%); partner: 20 (10.3%); other relative: 11 (5.7%)
Lives with patient?	Yes: 174 (89.7%)
Relationship characteristics:	
Mean weekly contact > 32 h	159 (82.0%)
Mean (SD) hours of personal contact	5.6 (1.1)
Mean (SD) no. days lived together in previous month; median	24.1 (10.1); 28.0

n = 194. Monthly net income is coded into six range scores in item 14 of the IEQ (1, minimum, to 6, maximum); hours of personal contact are coded into six range scores (1 = less than 1 h a week to 6 = more than 32 h a week).

### General data on the psychometrics of the IEQ Portuguese version

Factor analysis was conducted on this sample. According to Bartlett's test (value = 1,811.66 and *P *< 0.001) the correlation matrix was not an identity matrix and was therefore suitable for analysis. The KMO statistic was > 0.70 (0.798), the MSA values for all the individual items were > 0.60 (89% were > 0.70) and the absolute values of the off diagonal values were very low. Therefore, the analysis seemed appropriate. The factor analysis resulted in a very similar solution to the original one [5]. This solution is presented in Table 2 and it accounted for 47% of the total variance. The value of lost expected variance for IEQ-EU totals and subscales was acceptable (13%) if we complied with the original subscales, so we decided to use these in the remainder of the analysis.

**Table 2:** Principal component analysis on Involvement Evaluation Questionnaire (IEQ) items

Items	Factors			
	
	Tension	Supervision	Worrying	Urging
Percentage of variance (total = 46.55%)	24.95%	6.07%	8.10%	7.42%
How often during the past 4 weeks:				
(30) has the atmosphere been strained between both	0.786			
(32) been annoyed by relative's behavior	0.736			
(35) thought of moving out	0.7			
(31) has your relative caused a quarrel	0.679			
(34) felt threatened by your relative	**0.564**	**0.439**		
(27) carried out tasks normally done by your relative	0.49			
(33) heard from others they have been annoyed	0.478			
(16) have you encouraged your relative to take proper care				0.7
(18) to eat enough				0.64
(21) ensured that your relative has taken required medicines				0.615
(19) encouraged your relative to undertake some kind of activity				0.61
(17) helped your relative to take proper care of him/herself		**0.49**		**0.577**
(24) ensured your relative received sufficient sleep				0.554
(38) worried about the help/treatment your relative receives			**0.47**	**0.443**
(37) worried about your relative's safety				0.406
(40) have you worried about your relative managing financially			0.774	
(41) worried about your relative's future			0.727	
(42) worried about your own future			0.684	
(39) worried about your relative's general health			0.46	
(43) have your relative's mental health problems been a burden	**0.427**		**0.436**	
(26) have you guarded your relative from taking illegal drugs		0.713		
(25) guarded your relative from drinking too much alcohol		0.627		
(23) guarded your relative from self-inflicted harm		0.621		
(22) guarded your relative from committing dangerous acts		0.529		

Bold type indicates the factor to which the item was allocated in cases where it loaded ≥ 0.4 in two or more factors; items loading < 0.4 in any factor are not shown (20: accompanying on outside activities; 28: encouraging to get up in the morning; 29: sleep disturbance due to relative's behavior).

The distribution of IEQ-EU scales sum item scores and their internal consistency are presented in Table 3. Mean scores (SD), for each IEQ domain and total score, were: tension 0.88 (0.57); supervision 0.45 (0.58); worrying 2.3 (0.73); urging 1.07 (0.75); IEQ total score 1.12 (0.54). Overall, Cronbach α values were 'substantial' [7], being higher for the IEQ sum score (0.87) than for IEQ-EU subscales (0.71 to 0.74).

**Table 3:** Involvement Evaluation Questionnaire (IEQ) totals and domains: distribution of scores and internal consistency

	Sum item scores (0 to 4)			Cronbach's α	
	
	Minimum to maximum	Mean (95% CI) SEM	SD	Coefficient	95% CI (unilateral)
Tension	0 to 31 (0 to 36)	7.92 (7.19 to 8.64) 0.37	5.11	0.74	≥ 0.69
Supervision	0 to 21 (0 to 24)	2.70 (2.21 to 3.18) 0.25	3.45	0.71	≥ 0.65
Worrying	0 to 24 (0 to 24)	13.79 (13.04 to 14.54) 0.381	5.31	0.73	≥ 0.68
Urging	0 to 27 (0 to 32)	8.56 (7.71 to 9.41) 0.43	6.01	0.74	≥ 0.69
IEQ sum score	0 to 84 (0 to 108)	30.23 (28.16 to 32.31) 1.05	14.65	0.87	≥ 0.85

n = 194; minimum and maximum possible scores are given in brackets.

Tension and worrying, the two interpersonal domains, had a substantial correlation (r_S _= 0.71; *P *< 0.001), and there was a moderate coefficient between tension and urging (r_S _= 0.53; *P *< 0.001). All other correlations between domains were lower than 0.49, favoring the adopted original factor structure.

Test-retest reliability was assessed for 50 participants. The ICC for the IEQ-EU total score was 0.93 (95% CI 0.88 to 0.96, *P *< 0.001), while for subscales ICCs were: 0.88 (95% CI 0.79 to 0.93, P < 0.001) for tension, 0.79 (95% CI 0.63 to 0.88, *P *< 0.001) for supervision, 0.94 (95% CI 0.89 to 0.97, *P *< 0.001) for worrying, and 0.89 (95% CI 0.80 to 0.94, *P *< 0.001) for urging.

### Item-level results

Three IEQ-EU core module items which are not used to compute domain or total scores provided specific results on being able to pursue own activities and interests, getting used to mental illness, and self-perception of lack of coping ability. These three items were also scored from 0 (lower level) to 4 (higher level). Results (mean (SD)) were as follows: 1.5 (1.5) for pursuing own activities (median 1, meaning 'sometimes'); 1.9 (1.3) for getting used to problems (median 2, meaning 'fairly well'); and coping ability 1.9 (1.2) (median 2, meaning 'regularly felt able to cope').

### Ecological validity and acceptability of the IEQ-EU

The ecological validity of the IEQ Portuguese version is sufficient. Considering the FAPS baseline sample, the overall response rate was 100%, no questionnaires had to be discarded from analysis, and the applicability was also sufficient. However, caregivers varied in their ability to complete the IEQ-EU on their own, reflecting heterogeneity of literacy levels. In total 61 respondents (56.5%) were able to fully self-complete questionnaires, 32 (29.6%) required significant help, and 15 (13.9%) had to be interviewed due to limited literacy. According to interviewers' impressions, assessments were easily conducted and item formulation and understandability seemed adequate, even for illiterate respondents. All but five caregivers fully completed the IEQ.

## Discussion

This study replicates a part of the EPSILON study methodology for adequate testing of validity and reliability of the IEQ-EU in a South European setting (Portugal), with a considerable sample size. The IEQ had been originally developed in the north of Europe (The Netherlands).

Regarding patients' characteristics, they are typical of a clinical sample of chronic psychotic users in mental health outpatient public practices in Portugal [1,17]. The levels of psychopathological symptoms and global functioning of these users are similar to the ones described in previous studies on caregiving [5]. Mean disability levels are around the 'poor adjustment' DAS category, again in agreement with our assumption that the sample is probably representative of populations with chronic psychosis in Portuguese services [1]. Moreover, the corresponding caregivers' characteristics and caregiving arrangements are close to the usual pattern in South Mediterranean countries [1,5].

Overall, validity of the Portuguese IEQ-EU translation has been established. In this study, the ecological, face and content validity of the questionnaire were grounded on quality assurance of the translation, and on replication of the EPSILON methodology. Criterion and construct validity, and sensibility to change, were not directly approached here. However, the FAPS survey contributed to their specific testing in Portugal by comprehensively assessing 108 of these 194 patient-caregiver dyads, as reported elsewhere [18,19]. Construct validity is particularly sound [19] (Gonçalves-Pereira M, Xavier M, van Wijngaarden B, Papoila AL, Schene AH, Caldas de Almeida JM: Impact of psychosis in a Portuguese population: a cross-cultural exploration of burden, distress, positive aspects and clinical-functional correlates, unpublished).

Our data on psychometric properties ensure considerable reliability. Concerning internal consistency, alphas were slightly lower than in the EPSILON global sample, but some of them were more satisfactory than in some of the EPSILON centers [8]. Regardless of comparisons, they were 'substantial' according to the EPSILON convention [7] and 'good', according to Streiner and Norman, for this kind of scales [27]. ICCs on test-retest were invariably 'substantial' to 'high' [7]. We emphasize that the 50 respondents constituted a larger subsample for this kind of testing than most in the EPSILON study [8].

Our results also suggest that the original IEQ-EU factor structure can be preserved, as its use in our sample did not lead to too much loss of explained variance. Rank order of the four IEQ-EU domains was the same as in the EPSILON and previous Dutch studies: the mean score of worrying is the highest, followed by urging, interpersonal tension and supervision [5,21]. We consider this finding as another sign of validity.

Therefore, in Portugal, the IEQ-EU seems to cover the same caregiving domains that have been described for other countries, and instrument bias in the assessment of differences in caregiving consequences appears unlikely. In the FAPS baseline assessments, ecological validity of the IEQ-EU was evident, with high response rates and very few missing data.

### Limitations of the study

We did not use randomization procedures so sampling bias cannot be fully discarded. Therefore, despite the strong impression that our participants represent a typical sample of caregivers of chronic psychotic patients in Portugal, one must theoretically recognize limitations to the generalizability of our findings. We also did not use a semi structured psychiatric interview, although a robust clinical diagnosis ascertainment was ensured for the majority of cases.

All assessments were conducted in clinical settings, and postal means were not used. This may have positively influenced response rates and questionnaires' acceptability.

## Conclusions

A heavy negative impact of caregiving in severe mental illness has been acknowledged once more in a regional sample [28]. There is a need to continuously address vulnerable caregivers, and feasible risk assessment routines must be pursued.

The Portuguese version of the IEQ-EU is valid and reliable for research use, but its clinical usefulness remains a challenging topic. Regardless of this, the available evidence sustains the maintenance of the original IEQ factor structure in Portugal.

## Competing interests

The authors declare that they have no competing interests.

## Authors' contributions

MG-P conceived the present study, which was designed together with BvW, MX, JMCdA and AHS. MG-P coordinated the fieldwork and data analysis, and drafted the initial manuscript together with BvW. ALP supervised statistical procedures. All authors contributed significantly to the critical revision of the final manuscript.

## Authors' information

MG-P, MD PhD, is a psychiatrist and family therapist, and Professor of Medical Psychology in the Department of Mental Health, FCM, NOVA University, Lisbon. His interests include family issues in mental health. BvW, PhD, is senior research associate at the Trimbos-instituut, The Netherlands Institute of Mental Health and Addiction. He is one of the developers of the Involvement Evaluation Questionnaire. MX, MD, PhD, is Professor of Psychiatry and Mental Health in the Department of Mental Health, FCM, NOVA University, Lisbon. ALP, PhD, is a statistician in the Department of Biostatistics and Informatics, FCM, NOVA University, Lisbon. JMCdA, MD, PhD, is Full Professor of Psychiatry and Mental Health in the Department of Mental Health, FCM, NOVA University, Lisbon. He is also the Dean of the faculty. AHS, MD, PhD, is Professor of Psychiatry and head of the Program for Mood Disorders in the Department of Psychiatry, Academic Medical Center, University of Amsterdam, Amsterdam, The Netherlands.
